# Turn Around Freezing: Community-Living Turning Behavior in People with Parkinson’s Disease

**DOI:** 10.3389/fneur.2018.00018

**Published:** 2018-01-26

**Authors:** Martina Mancini, Aner Weiss, Talia Herman, Jeffrey M. Hausdorff

**Affiliations:** ^1^Department of Neurology, School of Medicine, Oregon Health & Science University, Portland, OR, United States; ^2^Center for the Study of Movement, Cognition and Mobility, Neurology Department, Tel Aviv Sourasky Medical Center, Tel Aviv, Israel; ^3^Department of Physical Therapy, Sackler Faculty of Medicine, Sagol School of Neuroscience, Tel Aviv, Israel; ^4^Alzheimer’s Disease Center, Department of Orthopaedic Surgery, Rush University Medical Center, Chicago, IL, United States

**Keywords:** freezing of gait, inertial measurement unit, Parkinson disease, community-living monitoring, turning movements

## Abstract

Difficulty in turning while walking is common among patients with Parkinson’s disease (PD). This difficulty often leads to significant disability, falls, and loss of function; moreover, turning is a common trigger for freezing of gait (FoG). We hypothesized that the quantity and quality of turning mobility while walking during daily life would be different among subjects with PD with and without FoG. Here, we investigated, for the first time, the turning quality during daily life as it relates to FoG in people with PD using a single inertial sensor. Ninety-four subjects with PD (among whom 25 had FoG) wore an inertial sensor attached by a belt on the lower back during normal daily activity consecutively for 3 days. An algorithm identified periods of walking and calculated the number and quality metrics of turning. Quality, but not the quantity, of turning at home was different in freezers compared to the non-freezers. The number of turns (19.3 ± 9.2/30 min in freezers, 22.4 ± 12.9/30 min non-freezers; *p* = 0.194) was similar in the two groups. Some aspects of quality of turns, specifically mean jerkiness, mean and variability of medio-lateral jerkiness were significantly higher (*p* < 0.05) in the freezers, compared to non-freezers. Interestingly, subjects with FoG showed specific turning differences in the turns with larger angles compared to those without FoG. These findings suggest that turning during daily activities among patients with PD is impaired in subjects with FoG, compared to subject without freezing. As such, clinical decision-making and rehabilitation assessment may benefit from measuring the quality of turning mobility during daily activities in PD.

## Introduction

Turning while walking, an essential part of mobility in daily life, is a challenging task that requires the control of dynamic balance. In fact, the majority of activities in the home require 3–4 turns and over 50% of daily steps are turning steps (1). Unfortunately, when a fall occurs during a turn, it is eight times more likely to result in a hip fracture, as compared to falls that occur in other situations. Often, the result is falling to the ground on the side and onto the greater trochanter of the hip (2). A recent video analysis of the most common circumstances of falls in daily life in elderly people residing in long-term care including people with Parkinson’s disease (PD) revealed that the individuals with PD were 1.3 times as likely as those without PD to fall because of incorrect weight shifting, which is also needed for turning (3).

The majority of people with PD have difficulty in turning, even in the early stages of the disease (4), likely because of the complex interaction of gait with dynamic balance during turning (1). Turning in PD is characterized by slow speed, long turning duration, large number of steps (5–7), a narrow base of support (8), impaired segmental coordination of rotation (“en-bloc”) (9–11), and decreased postural stability, particularly during fast locomotion (12). It is not surprising, therefore, that people with PD fall five times more than age-matched older adults and they often fall while turning (3).

Difficulty in turning occurs among patients with PD, in general, and, more specifically, turning is known as one of the most common conditions to provoke freezing of gait (FoG), defined as the inability to generate effective forward stepping movements (13, 14). In addition, sharper and, therefore, more asymmetric turns, are associated with increased step time variability and more freezing episodes in subjects with PD who experience FoG (15, 16). FoG in subjects with PD is an environmentally sensitive, intermittent problem that mostly occurs during postural transitions, such as turning (13, 17), or in challenging situations (e.g., dual tasking, crowded spaces), greatly affecting mobility during daily life. Although patients may report that they have FoG, it often cannot be observed during clinical appointments or in the laboratory (17–19). This might be because FoG is triggered in specific conditions, such as multi-tasking in challenging environments. However, the impact that FoG poses in everyday mobility and the ability to turn during community ambulation has not yet been well-studied in PD patients who experience FoG.

A growing body of literature supports the application of wearable, light-weight inertial sensors to characterize mobility outside the laboratory setting (20–28), for example, at home or in the community in general, and in PD, in particular. When measuring mobility in the home environment, it is important to consider the added value of “quantity” (e.g., number of turns or steps) and “quality” measures. Quality measures have shown to be more informative compared to the quantity of mobility (19–27). By quantity-related measures, we refer to the overall amount of movement during daily life (e.g., the number of steps, number of turns, walking bouts, time spent in different activities), while by quality-related measures we refer to the properties of those activities (e.g., gait consistency, variability, turning velocity, smoothness). To date, one study used body-worn sensors to compare daily mobility in subjects with PD with and without FoG over multiple days (29). Quantity and quality measures were obtained by continuously monitoring gait over 3 days with an inertial sensor on the lower back. Interestingly, freezers showed similar *quantity* of gait but different *quality of gait*, particularly, altered gait variability and inconsistency during spontaneous community ambulation. However, this work was limited to walking performance, and, thus far, little is known about whether turning behavior differs between people with and without FoG during community ambulation.

To better understand the impact of FoG on everyday mobility, in the present study, we investigated whether turning in the daily living home environment in subjects with PD who experience FoG is more impaired than in subjects without FoG. We hypothesized that both quantity and quality measures of turning would be worse in PD with FoG, as compared to PD subjects who do not experience FoG, since patients who experience FoG might avoid turns that elicit freezing. We also investigated the association between turning and disease severity, hypothesizing that progression of the disease will lead to more marked turning alterations.

## Materials and Methods

### Participants

Participants who attended the outpatient clinic of the Movement Disorders Unit of the Tel Aviv Sourasky Medical Center and from other affiliated clinics were studied. Full details of the recruitment procedures are provided in Weiss et al (30). Briefly, subjects were included if they were diagnosed by a movement disorders specialist and fullfilled the UK PD society Brain Bank criteria. Other inclusion criteria were: age (between 40 and 85 years old), a Mini–Mental State Examination (MMSE) score >24, and ability to walk independently. All the participants provided informed written consent approved by the human studies committee of the Tel Aviv Medical Center.

### Clinical Assessment

All the participants were clinically rated based on the Unified PD Rating Scale (MDS-UPDRS) (31). The new freezing of gait questionnaire (NFOG-Q) was used to assess the presence of FoG (14), specifically individuals were classified as freezers if they had experienced freezing episodes during the past month (Part I of NFOG-Q). The participants were further evaluated with performance-based measures such as the Activity Specific Balance confidence scale and gait speed during usual walking (29).

### Community Ambulation Protocol and Data Analysis

**Figure 1:**
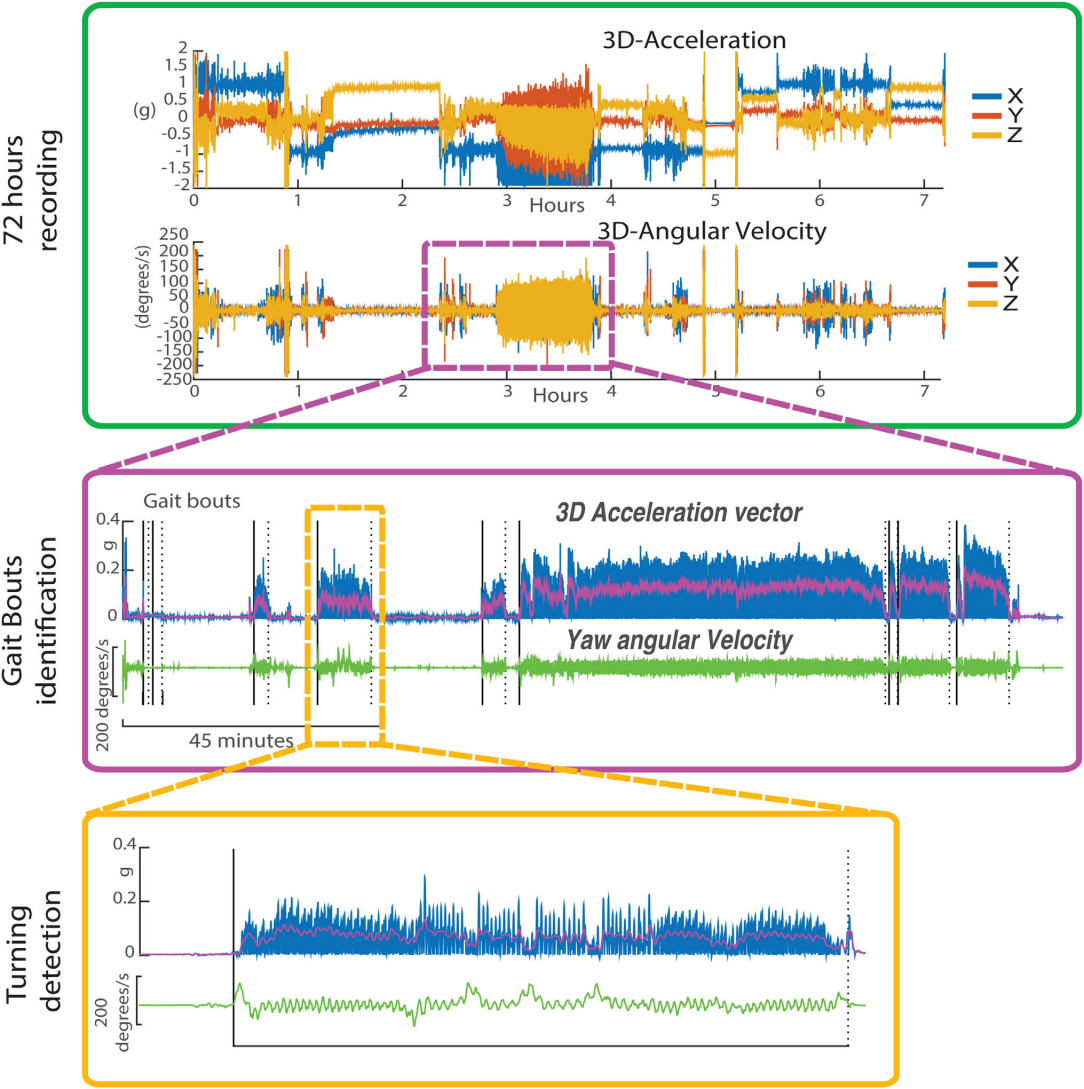
Profile of the inertial sensor data worn on the waist for 72 h. Upper panel: 3D acceleration and 3D angular velocity from continuous monitoring over 7 h. Middle panel: details on gait bouts identification. Lower panel: detailed profile on the Yaw angular velocity during a single gait bout.

As previously described (25, 26, 28), participants wore a small, light-weight sensor (McRoberts, DynaPort Hybrid system, The Netherlands), including a triaxial accelerometer and a triaxial gyroscope, attached to a belt on their lower back for three consecutive days (except during activities like showering or swimming). Data were recorded at 100 Hz and stored in the secure digital card of the monitor, and later transferred to a laptop for analysis. The steps of the algorithm steps are summarized in Figure 1. We validated a similar algorithm (using a different inertial sensor) and a Motion Analysis System (Santa Rosa, CA, USA) in a previous study in the Balance Disorders Laboratory at the Oregon Health and Science University in 15 subjects with PD and 19 age-matched control subjects. Compared to Motion Analysis, the algorithm maintained a sensitivity of 0.90 and a specificity of 0.75 for detecting turns. More details about the algorithm development and its validity can be found in El-Gohary et al. (32). Briefly, from the data collected over 72 h, periods of walking (32) were first detected, from the angular velocities and accelerations, in windows of 30 min. Specifically, walking periods of 10 s or longer were defined as “gait bouts” (from the triaxial angular velocities). By doing so, we excluded “gait bouts” which likely had FoG, which are typically shorter, since our goal here was to investigate quality of turns free from FoG. Then, the algorithm searched for potential turns within each gait bout. We used the horizontal rotational rate of the lumbar sensor (yaw) to detect turning events during gait bouts. Specifically, a turn was defined as a trunk rotation around the vertical plane with a minimum of 40°. Only turns with durations between 0.5 and 10 s, and turn angles of 40° or more were considered. Relative turn angles were obtained by integrating the angular rate of the lumbar sensor about the vertical axis.

The mean and coefficient of variation (CV) were calculated across the 72 h for the following measures that were determined to assess different aspects of the amount and quality of turns: (1) number of turns per 30 min, (2) turn angle amplitude (degrees), (3) turn duration (seconds), (4) mean and peak turn velocity (degrees/seconds), (5) turn jerkiness, calculated as the derivative of the acceleration is a proxy for the fluidity of turning [m^2^/s^5^, 2D, i.e., combining antero-posterior and medio-lateral acceleration and only medio-lateral (33)], and (6) turn medio-lateral range of acceleration (m/s^2^).

### Statistical Analysis

A univariate, general linear model was used to determine whether differences in the continuous monitoring turning measures existed among the two groups (i.e., freezers and non-freezers). By visually inspecting the distribution of angle amplitude, we determined the 120° cutoff to balance evenly the number of turns in the two categories. Therefore, a repeated measures, general linear model was carried out to investigate the effects of angle amplitude, small-to-medium (>40° and <120°) vs. medium-large (>120° and <260°), group (i.e., freezers and non-freezers), and group × angle amplitude interaction. To minimize the bias of unbalanced groups (25 vs. 69), we used a Type II instead of Type III sum of squares for both analyses (34). In addition, disease duration and gait speed were used as covariates in order to adjust for any group differences in these subject characteristics. Finally, Pearson correlation coefficients were used to assess the relationships between turning metrics and clinical tests. All the analyses were performed using SPSStatistics Software (IBM, v23) and Matlab (R2016 b, Mathworks).

## Results

Based on the NFOG-Q, 25 subjects were classified as freezers and 69 as non-freezers (see Table 1). Subjects in the two groups were similar with respect to age, MMSE, and UPDRS Part III. However, freezers showed longer disease duration, higher Hoehn and Yahr stage, and slower gait speed. Therefore, the analyses on turning measures were carried out with disease duration and gait speed as covariates.

**Table 1:** Subjects characteristics in Parkinson’s disease (PD) freezers and PD non-freezers.

	Non-freezers *N* = 69	Freezers *N* = 25	*p*-Value
Age (years)	65.4 ± 9.7	64.2 ± 8.8	0.10
Gender (% female)	27%	12%	0.094
Disease duration (years)	4.9 ± 2.8	7.6 ± 4.4	0.001
Mini–Mental State Examination	28.9 ± 1.1	28.4 ± 2.2	0.16
New freezing of gait questionnaire	0 ± 0	16.2 ± 7.4	<0.0001
Hoehn and Yahr stage	2.4 ± 0.5	3.2 ± 0.8	0.001
UPDRS Part III (ON)	33.4 ± 12.3	36.4 ± 12.4	0.33
Activities-specific Balance Confidence Scale (%)	89.0 ± 13.4	72.8 ± 19.8	0.001
Gait speed OFF (m/s)	1.14 ± 0.20	1.04 ± 0.22	0.035

### Quantity and Quality of Turning

Quantity of turning, assessed as the mean number of turns/30 min, was similar in freezers and non-freezers (*F* = 1.821, *p* = 0.181), see Table 2. In contrast, certain aspects of the quality of turns, specifically mean jerkiness, mean and cv of medio-lateral jerkiness were significantly higher in the freezers compared to the non-freezers (*F* = 5.636, *p* = 0.020; *F* = 6.249, *p* = 0.014; *F* = 4.606, *p* = 0.035, respectively). The mean turn angle was significantly smaller in freezers compared to non-freezers (*F* = 5.850, *p* = 0.018). The freezing severity, as assessed by the NFOG-Q, was significantly associated with the mean turn angle (*p* = −0.59, *p* = 0.001) in the freezers group, specifically, freezers who showed lower (i.e., better) scores on the NFOG-Q tended to have higher mean turn angles.

**Table 2:** Three-day measures of quantity and quality of turning in non-freezers and freezers.

Measure	Non-freezers *N* = 69	Freezers *N* = 25	Corrected model	
	Mean ± STD	Mean ± STD	*F*-value	*p*-value
**Quantity**				
*N* turns/30 min	19.3 ± 9.2	22.4 ± 12.9	1.821	0.181
**Quality**				
Turn angle (degrees)	92.5 ± 5.3	89.0 ± 9.2	**5.850**	**0.018**
Coefficient of variation (CV) turn angle	0.40 ± 0.05	0.39 ± 0.05	2.713	0.103
Turn duration (s)	2.4 ± 0.3	2.2 ± 0.4	3.718	0.072
CV turn duration	0.42 ± 0.05	0.43 ± 0.03	0.611	0.437
Mean velocity (degrees/s)	34.4 ± 4.3	35.7 ± 6.5	1.057	0.307
CV mean velocity	0.26 ± 0.03	0.26 ± 0.04	1.769	0.187
Peak velocity (degrees/s)	67.0 ± 8.9	70.0 ± 13.4	1.359	0.247
CV peak velocity	0.28 ± 0.03	0.27 ± 0.04	0.278	0.600
2D jerk (m^2^/s^5^)	10.2 ± 1.2	10.9 ± 1.6	**5.636**	**0.020**
CV 2D jerk	0.32 ± 0.06	0.33 ± 0.07	3.721	0.057
ML jerk (m^2^/s^5^)	0.29 ± 0.09	0.37 ± 0.14	**6.249**	**0.014**
CV ML jerk	0.63 ± 0.11	0.66 ± 0.15	**4.606**	**0.035**
ML range (m^2^/s)	0.27 ± 0.04	0.29 ± 0.05	1.491	0.225
CV ML range	0.29 ± 0.06	0.30 ± 0.08	1.533	0.219

*Disease duration and gait speed are covariates in all of the analyses in this table; bold values indicate when p < 0.05*.

### Turning with Larger Turn Amplitude Is Different in Freezers Compared to Non-freezers

Due to the difference in mean turn angle between the groups, we separated turning characteristics for small-medium (>40° and <120°) turn angle amplitude vs. medium-large turn angle amplitude (>120° and <260°). 100% of freezers and 100% of non-freezers showed small angle amplitude turns over the 3 days of monitoring. Similarly, 84% of the freezers and 94% of the non-freezers showed the presence of larger amplitude turns.

**Figure 2:**
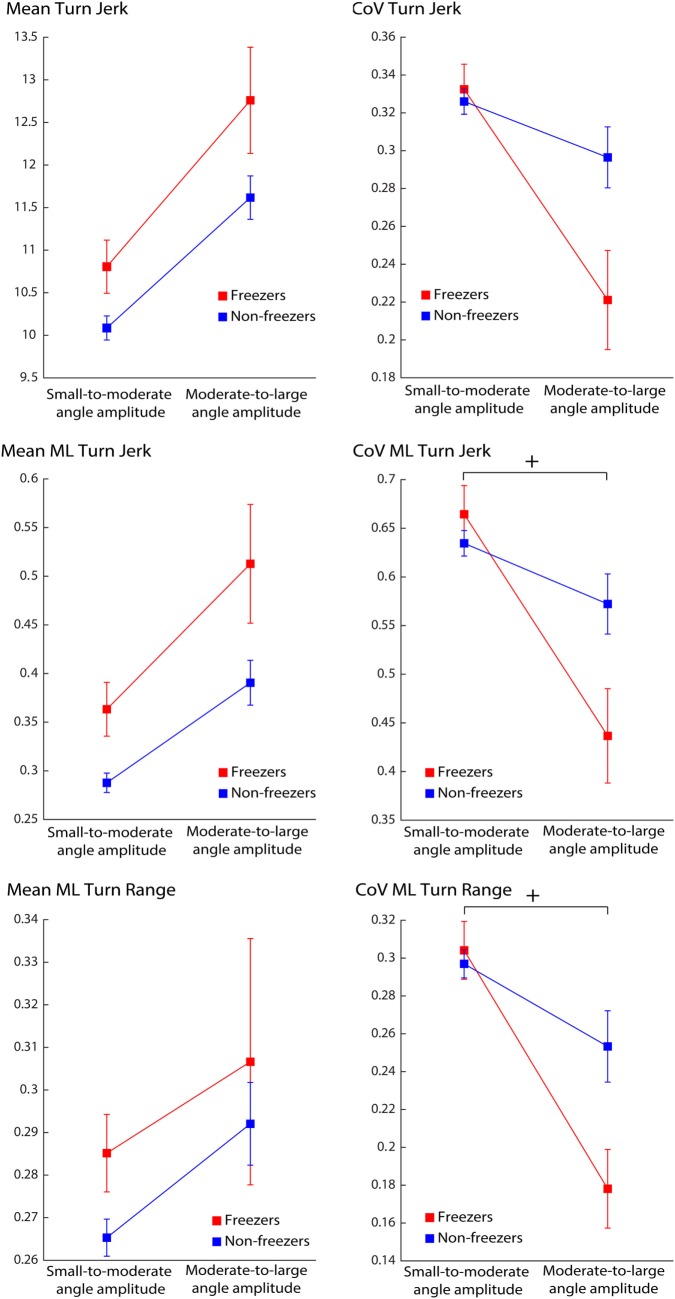
Effect of turning angle amplitude in Parkinson’s disease freezers and non-freezers on quality of turns over 72 h. Legend: ^+^significant interaction effect, *p* < 0.05.

Mean turn duration was different across angle amplitude, but did not show a significant group or group × angle amplitude interaction effect (Table 3). Interestingly, only the variability of medio-lateral jerk and medio-lateral range showed a significant group × angle interaction effect (Figure 2). Mean jerk and mean medio-lateral jerk during turning almost approached significance for group effects (Table 3).

**Table 3:** Three-day measures of quality of turning divided by angle amplitude in non-freezers and freezers.

Measure	Angle type	Non-freezers *N* = 69	Freezers *N* = 25	Corrected model					
				Group		Angle		Interaction	
				
		Mean ± STD	Mean ± STD	*F*-value	*p*-value	*F*-value	*p*-value	*F*-value	*p*-value
Turn duration	S	2.347 ± 0.317	2.158 ± 0.372	1.915	0.170	**9.97**	**0.002**	0.010	0.921
	M	3.042 ± 0.810	2.819 ± 0.694						
Coefficient of variation (CV) turn duration	S	0.423 ± 0.042	0.430 ± 0.030	0.103	0.749	0.629	0.430	0.436	0.511
	M	0.392 ± 0.164	0.358 ± 0.216						
Mean velocity	S	34.161 ± 4.329	35.587 ± 6.398	1.189	0.279	1.022	0.315	0.345	0.559
	M	36.471 ± 5.825	38.008 ± 5.779						
CV mean velocity	S	0.266 ± 0.035	0.265 ± 0.039	0.221	0.639	0.201	0.655	0.221	0.639
	M	0.203 ± 0.083	0.204 ± 0.105						
Peak Velocity	S	66.158 ± 8.625	69.526 ± 12.902	1.749	0.190	1.576	0.213	1.654	0.202
	M	73.556 ± 13.206	78.563 ± 16.217						
CV peak velocity	S	0.280 ± 0.034	0.273 ± 0.039	0.131	0.719	0.076	0.784	0.306	0.582
	M	0.235 ± 0.104	0.206 ± 0.096						
2D jerk	S	10.088 ± 1.192	10.806 ± 1.559	3.137	0.080	0.840	0.362	0.193	0.661
	M	11.617 ± 2.133	12.760 ± 3.118						
CV 2D jerk	S	0.325 ± 0.053	0.332 ± 0.066	0.499	0.482	0.487	0.487	3.868	0.053
	M	0.301 ± 0.132	0.221 ± 0.131						
ML jerk	S	0.288 ± 0.083	0.368 ± 0.139	3.192	0.078	1.873	0.175	0.196	0.659
	M	0.386 ± 0.191	0.513 ± 0.305						
CV ML jerk	S	0.630 ± 0.105	0.664 ± 0.147	0.247	0.621	1.000	0.320	**4.779**	**0.032**
	M	0.572 ± 0.259	0.437 ± 0.243						
ML range	S	0.266 ± 0.036	0.285 ± 0.046	0.115	0.735	0.576	0.450	0.552	0.460
	M	0.292 ± 0.082	0.307 ± 0.145						
CV ML range	S	0.296 ± 0.062	0.304 ± 0.076	1.841	0.179	0.219	0.641	**5.938**	**0.017**
	M	0.253 ± 0.158	0.178 ± 0.104						

*S, small-medium turn angle amplitude; M, medium-large turn angle amplitude*.

*Disease duration and gait speed are covariates in all of the analyses in this table; bold values indicate when p < 0.05*.

### Quantity and Quality of Turning While Walking Are Associated with Disease Severity

**Figure 3:**
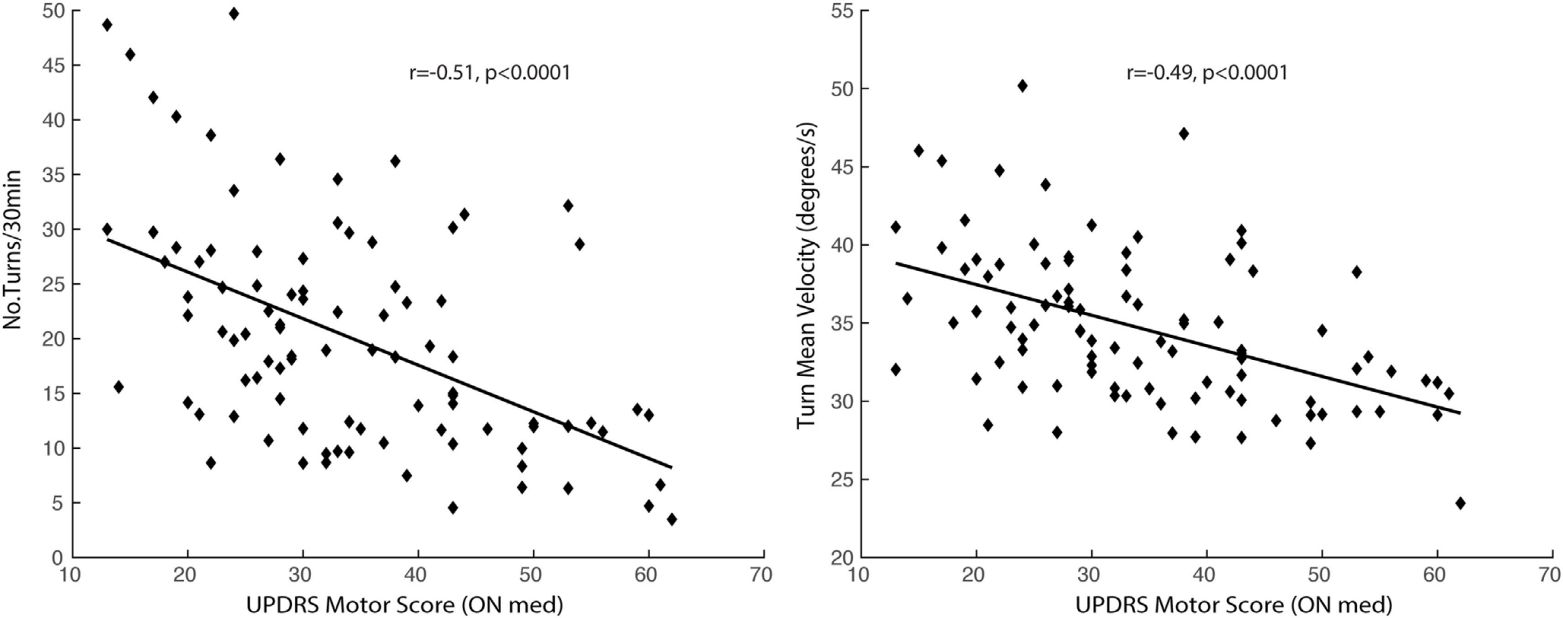
Association between disease severity, as measured by the UPDRS Motor Score, and quantity and quality of turning measured over 72 h.

**Figure 4:**
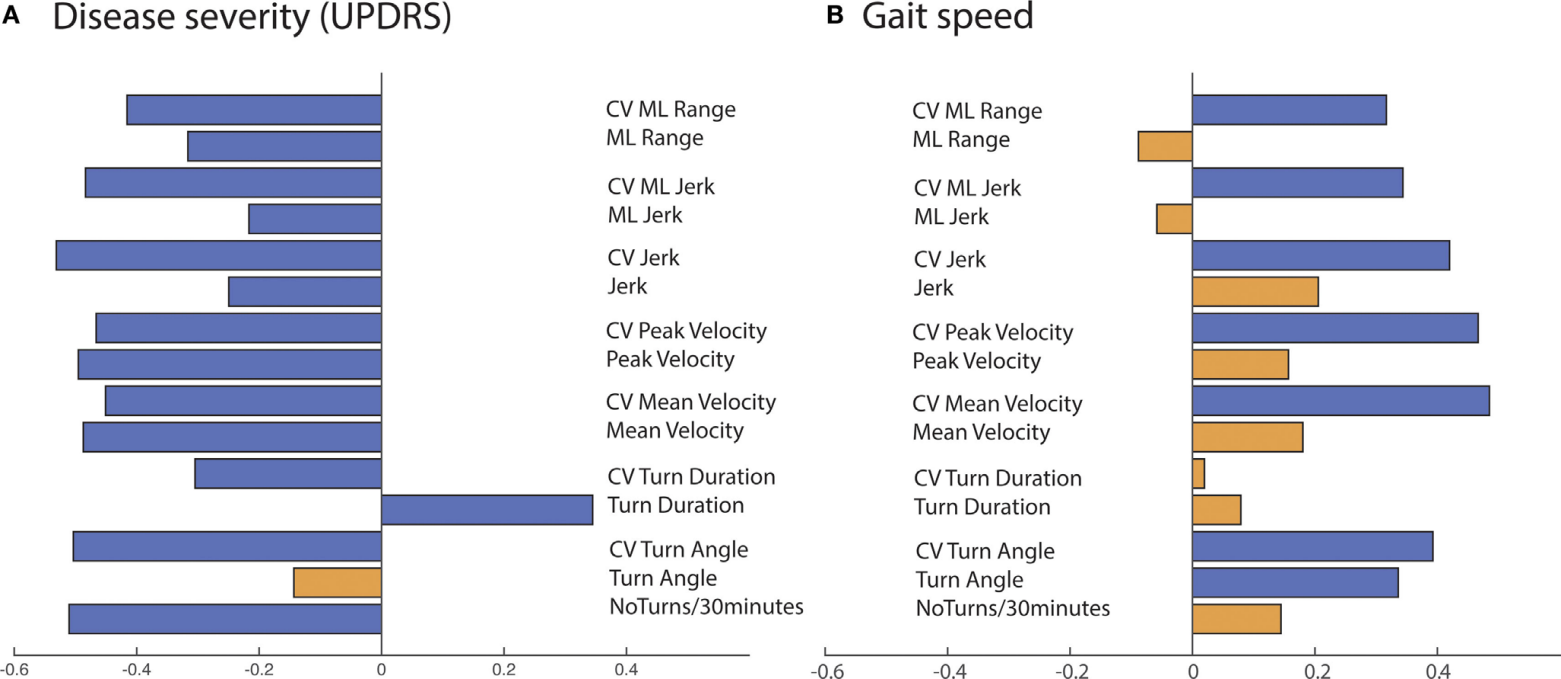
Summary of Pearson’s correlations of disease severity **(A)** and gait speed **(B)** with quantity/quality of turning over 72 h across all the Parkinson’s disease participants. Blue bar: *p*-value < 0.05, orange bar: *p*-value > 0.05.

Measures of the quantity and quality of turning, except for mean turn angle, were significantly associated with disease severity, as measured by the MDS-UPDRS Part III (ON medication) (see Figures 3 and 4A). The variability of all the quality turning measures and turn angle were associated with gait speed, as measured in the lab in the ON state (Figure 4B). Specifically, subjects with larger turning variability had higher gait speeds.

## Discussion

Our findings show evidence that certain aspects of turning during daily activities are different in people with PD who experience freezing of gait, compared to those who do not. Specifically, we showed that quality, but not quantity of turning differed between PD freezers and PD non-freezers, particularly at the largest turning angles.

Contrary to our initial hypothesis of impaired quantity and quality of turns in freezers compared to non-freezers, we were surprised to find that the number of turns/30 min was similar in the two groups. In hindsight, perhaps the absence of a group difference is not totally unexpected. Previous work reported that the number of steps during community ambulation was similar in PD freezers and PD non-freezers (29). One could suggest that the more someone walks during daily living, the more likely that the number of turns will increase during ambulation. Perhaps, this explains why freezers and non-freezers had a similar amount of turns. Regardless of the exact explanation, this finding suggests that freezers did not try to avoid turns during daily living, even though they are known to provoke FoG.

Although a similar number of turns were found in both groups, the freezers showed significant alterations in certain aspects of the quality of turns compared to the non-freezers. When considering all the angles together, freezers showed a smaller turning angle, higher mean jerk (2D and medio-lateral), and higher ML jerk variability. When separating turning characteristics by angle amplitude, only jerk and medio-lateral jerk during turning showed a trend in approaching significant group effects, suggesting the presence of higher jerkiness during turning in the freezers group, compared to the non-freezers. Interestingly, the variability characteristics of medio-lateral range and medio-lateral jerk showed a significant group by angle interaction effect, with freezers displaying lower variability characteristics for larger amplitude turn angles, compared to smaller amplitude angles.

The finding of smaller turning angle in freezers could indicate a tendency of avoiding larger turning angles to potentially avoid freezing episodes or because freezers circumvent a variety of larger turns due to a lack of skills necessary to maintain medio-lateral balance during sharper turns. In fact, several reports indicate that freezing episodes are more likely to be elicited by larger turning amplitudes (13, 35, 36). The higher jerkiness could reflect either an increased number of steps to complete the turn or an increased amount of trunk corrections while turning in freezers, compared to non-freezers. Moreover, the higher jerk variability could indicate a more variable turning strategy (when merging all turning angles together). The possible increased amount of trunk corrections could be due to an abnormal lateral anticipatory postural adjustment to unload the stepping leg, consistent with earlier findings showing a high association between weight shifting difficulties in the medio-lateral direction and freezing severity during a repetitive stepping in place task (37). Alternatively, it could be a direct consequence of a more pronounced impairment in the craniocaudal sequence preceding and during turning as recently reported in freezers compared to non-freezers (14). A potentially delayed or abnormal preparation for the change in walking direction could result in more adjustment during turning and, therefore, a higher jerk. The lower variability found in freezers for larger angles could indicate a tendency to repeat over and over the same turning strategy for larger turning amplitudes in order to potentially avoid freezing episodes.

Additionally, a previous analysis of the same cohort (29) characterized community-living gait during 72 h of monitoring. The freezers showed higher variability and less consistency of gait compared to the non-freezers. The increased gait variability in the home environment is in line with findings from several studies conducted in the laboratory setting that reported higher gait variability among freezers in situations that are known to provoke FoG (e.g., prior to turns, while dual tasking) (13, 38–40). These findings support the idea that freezers have increased gait variability while walking in between FoG episodes. Overall, combining the turning and gait findings, we can speculate that the increase in gait variability prior to FoG provoking situations combined with a motor task requiring more medio-lateral control of balance (such as turning) may contribute to an overload of the system that results in FoG episodes (13, 41).

The association between turning characteristics measured during daily life and disease severity confirms the findings of a previous feasibility study recently published in 13 subjects with PD of mild-to-moderate severity (UPDRS Motor Score of 24.5 ± 7.5) and 19 healthy controls of similar age (42). Here, in a much larger cohort of 94 subjects in a moderate-to-severe stage of disease (UPDRS Motor Score ranging from 13 to 62), we observed that reduced number of turns, longer turn duration, reduced turn velocity, reduced turn jerkiness, and range were associated with disease progression. Interestingly, the variability characteristics of the turning metrics showed a negative association with disease severity, meaning that variability of quality of turns tended to decrease with disease progression, potentially resulting from a less variable, “en-bloc” turning strategy. In addition, only the variability of turn quality and the turning angle were associated with gait speed, i.e., a faster gait was associated with a larger turn variability and larger turn angle amplitude. Although this might seem counterintuitive, it is in keeping with the concept introduced by Brach et al. (43), suggesting that either too much or too little variability can be associated with impaired mobility. Translating this concept to our findings on turning, too much variability could reflect imbalance or compensation for impaired postural control while turning, whereas too little variability could be associated with a loss of skills necessary to adapt postural control for a variety of turns. Further studies are needed to fully understand the relationships between these possible mechanisms and FoG and why only certain aspects of turning quality differ in freezers as compared to non-freezers.

This study has several strengths and limitations. Advantages include the relatively large number of participants with PD who underwent continuous monitoring of daily life activities over 72 h. However, only 25 out of 69 subjects reported FoG, and although this was accounted for in the statistical analysis, a more balanced number of subjects in each group would have been ideal. The small, light-weight sensor on the lower back allowed monitoring mobility function in an unobtrusive way. In this study, we did not aim to measure FoG itself or falls, a possible consequence of FoG, but rather characterize turns during walking in patients with and without FoG. In addition, by evaluating only gait bouts of 10 s and longer, our algorithm excluded periods of FoG; however, we cannot rule out that very short periods of hesitation were incorporated in the analysis, as the accelerations detected at the lumbar level are very likely attenuated compared to those detected on the lower legs. In the future, it would be helpful to identify and objectively characterize the impact of freezing during the day (i.e., measuring total time spent freezing, its severity and relationship to any falls) to allow for a more comprehensive quantitative rehabilitation outcome. Moreover, the cross-sectional nature of the study limits the ability to interpret the results on disease progression; a longitudinal study is better suited for that purpose. Still, the present findings suggest that a single inertial measurement unit that captures turning quality and quantity during daily living may help to characterize the mobility of PD patients with and without freezing of gait.

## Author Contributions

JH and TH designed the work. TH and AW performed data acquisition. MM analyzed data. MM, AW, TH, and JH contributed to the interpretation of the data. MM drafted the manuscript. AW, TH, and JH participated in the critical revision process. All the authors approved the manuscript.

## Conflict of Interest Statement

The authors declare that the research was conducted in the absence of any commercial or financial relationships that could be construed as a potential conflict of interest.
